# Reconstructive Endovascular Treatment of Basilar Trunk and Vertebrobasilar Junction Aneurysms: A Review of 77 Consecutive Cases

**DOI:** 10.3389/fneur.2022.885776

**Published:** 2022-05-12

**Authors:** Qichen Peng, Yangyang Zhou, Wenqiang Li, Chao Wang, Linggen Dong, Shiqing Mu, Yisen Zhang

**Affiliations:** ^1^Department of Interventional Neuroradiology, Beijing Neurosurgical Institute, Beijing Tiantan Hospital, Capital Medical University, Beijing, China; ^2^Department of Neurosurgery, The First Affiliated Hospital of Zhengzhou University, Zhengzhou, China

**Keywords:** intracranial aneurysm, basilar trunk, vertebrobasilar junction, endovascular treatment, prognosis

## Abstract

**Background:**

Basilar trunk and vertebrobasilar junction (BTVBJ) aneurysms have a poor prognosis and are challenging to treat.

**Objective:**

This study aimed to evaluate the efficacy of reconstructive endovascular treatment for BTVBJ aneurysms and explore a treatment selection paradigm.

**Methods:**

Clinical and angiographic data from 77 patients with 80 BTVBJ aneurysms who underwent endovascular treatment with flow diverters (FDs) or conventional stent-assisted coiling between January 2016 and December 2020 were retrospectively analyzed. Aneurysm characteristics and treatment outcomes were compared between treatment groups.

**Results:**

Among the 77 study patients, 34 (44.2%) were treated with FDs and 43 (55.8%) with conventional stent-assisted coiling. Overall, 72.7% of patients achieved favorable clinical outcome at follow-up. The rate of procedure-related complications was 23.4%. The aneurysm occlusion rate at last follow-up did not differ between the FD and conventional stent groups (79.2% vs. 77.1%, *p* = 0.854). Although the occlusion rate immediately after the procedure was lower in the FD group (29.4%), incidence of progressive occlusion was significantly higher (62.5 vs. 5.7%; *p* < 0.001). The proportion of patients with large and giant aneurysms (≥10 mm) was significantly higher in the FD group (70.6 vs. 34.8%; *p* = 0.002). In patients with large or giant aneurysms, favorable clinical outcome at last follow-up was achieved in 75% of patients in the FD group but only 43.8% of patients in the conventional stent group (*p* = 0.046). Moreover, the complication rate was lower in the FD group, but the difference was not significant (20.8 vs. 37.5%; *p* = 0.247). The same analyses were performed for patients with small aneurysms (<10 mm) but no significant differences between the two groups were observed.

**Conclusion:**

Endovascular treatment of small BTVBJ aneurysms using either FDs or conventional stents was feasible and effective. In patients with large or giant aneurysms, treatment using FDs achieved higher rates of occlusion and favorable clinical outcome at last follow-up than conventional stent-assisted coiling.

## Introduction

Aneurysms of the basilar trunk and vertebrobasilar junction (BTVBJ) are rare, comprising only 2.7% of all intracranial aneurysms (1). According to the International Study of Unruptured Intracranial Aneurysms, posterior circulation aneurysms, particularly those located on the basilar artery, have a higher risk of rupture (2). Considering their high rupture risk and the potentially fatal consequences of rupture, treatment of these aneurysms is necessary. Surgery of posterior circulation aneurysms is difficult and risky because of their anatomical location; therefore, endovascular therapy has become the mainstay of treatment (3–6). Treatment using conventional stents and flow diverters (FDs) has been shown to be safe and effective (7–9). The use of FDs to treat posterior circulation aneurysms has become more common in recent years (10). This study aimed to evaluate the efficacy of reconstructive endovascular treatment (EVT) for BTVBJ aneurysms and explore a treatment selection paradigm.

## Materials and Methods

### Patient Selection and Data Collection

**Figure 1:**
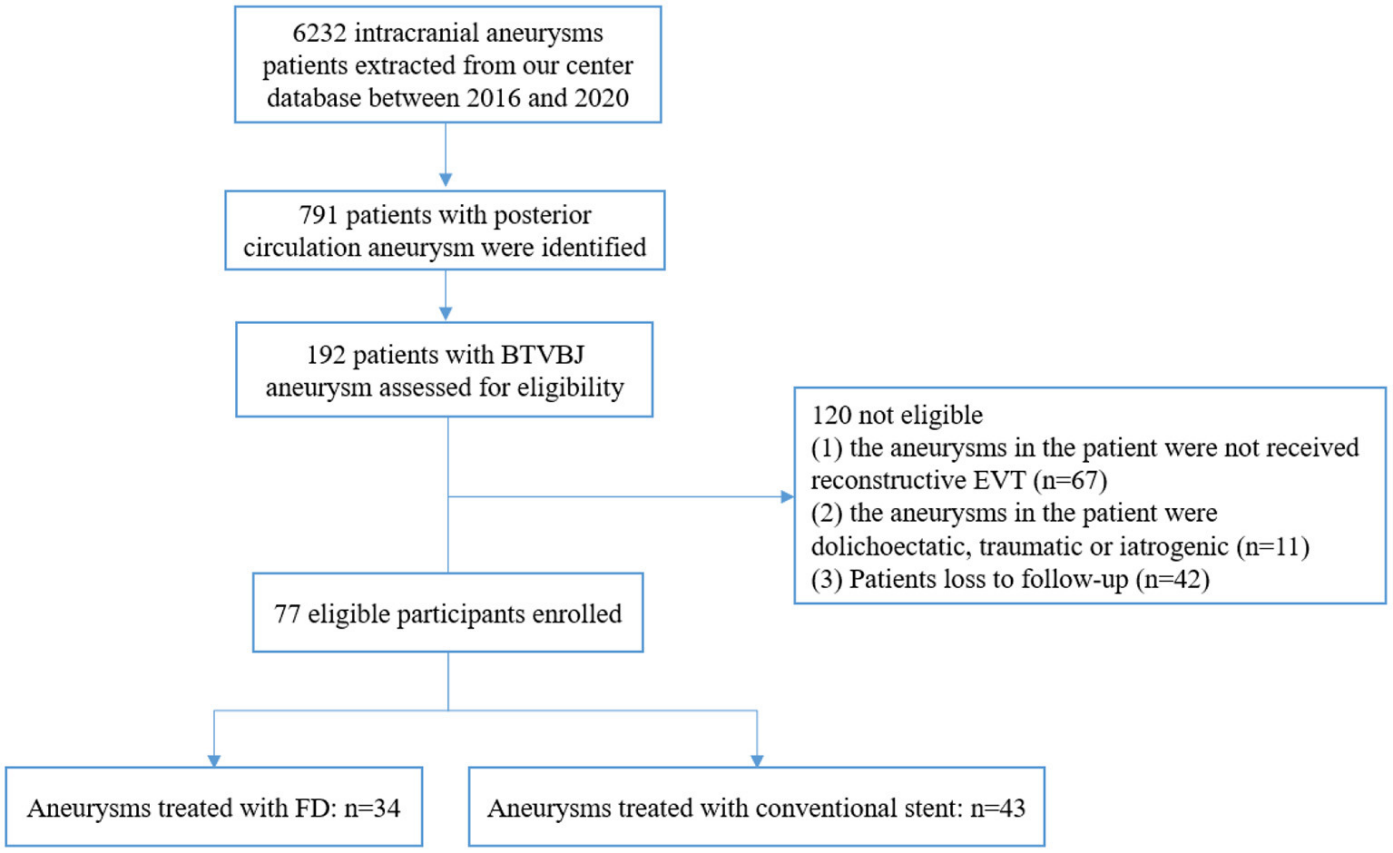
Study flowchart. BTVBJ, basilar trunk and vertebrobasilar junction; EVT, endovascular treatment; FD, flow diverter.

We retrospectively reviewed the data of patients with intracranial aneurysms who were treated in our center from January 2016 to December 2020. Patients who met the following criteria were included for analysis: (1) BTVBJ aneurysm was diagnosed using digital subtraction angiography (DSA); (2) the aneurysm was not dolichoectatic, traumatic or iatrogenic; (3) the aneurysm was treated using stent-assisted coiling or flow diversion; and (4) clinical follow-up data were available. The study flowchart is shown in Figure 1. BTVBJ aneurysm was defined as an aneurysm located anywhere from and including the vertebrobasilar junction to the superior cerebellar artery. Data including age, sex, smoking and alcohol use history, history of hypertension and diabetes mellitus, clinical profile, aneurysm characteristics, and procedural details and complications were obtained from the medical records. Imaging studies were examined to determine aneurysm number, size, shape, and parent artery. Approval by the institutional review board and ethics committee of Beijing Tiantan Hospital was obtained.

### Antiplatelet and Anticoagulation Therapies

Patients with an unruptured aneurysm received clopidogrel 75 mg and aspirin 100 mg each day for at least 5 days before the procedure. Those with a ruptured aneurysm received clopidogrel 300 mg and aspirin 300 mg 2 h before the procedure. Systemic intravenous heparin was administered throughout the endovascular procedure to maintain an activated clotting time between 250 and 300 s. Patients were treated with conventional stenting or flow diversion as appropriate. After the procedure, patients who underwent FD placement received clopidogrel for 6 months and aspirin indefinitely; those treated with conventional stenting received clopidogrel for 3 months and aspirin for 6 months.

### Endovascular Procedure

Procedures were performed by multiple neurointerventionalists. Treatment decisions were discussed among the neurointerventionalists and made by consensus at the daily peer-reviewed endovascular conference in our center. General anesthesia was used in all patients. After using the Seldinger technique to puncture the femoral arteries bilaterally, the sheath was placed. The guiding catheter was placed in the vertebral artery at the C1–C2 level for three-dimensional rotational DSA to select the best working projection and measure the parameters of the parent artery. Stent selection was at the discretion of the operator and was partly based on the results of DSA. In most cases, only implantation of a single stent or FD was required. Multiple devices were used in patients with long lesions or large aneurysms. The conventional stents used in this study included the Enterprise (Cerenovus, Raynham, MA, USA), LVIS (MicroVention, Tustin, CA, USA), Neuroform (Stryker Neurovascular, Fremont, CA, USA), and Solitaire (Covidien, Irvine, CA, USA) stents. The FD used was the Pipeline Embolization Device (Medtronic, Minneapolis, MN, USA). For aneurysms treated with flow diversion, the stent-jailing technique was used to coil the aneurysm or eccentric lumen if the diameter of the aneurysm or eccentric lumen exceeded 10 mm.

### Follow-Up and Clinical Outcomes

Follow-up data were obtained from the medical records and via telephone. Two experienced neurologists performed clinical evaluations and follow-up assessments. The modified Rankin Scale (mRS) was used to evaluate patients at hospital admission, discharge, and last follow-up. Favorable clinical outcome was defined as mRS score 0–2. Postoperative imaging follow-up was performed using DSA, computed tomography angiography (CTA), and magnetic resonance angiography (MRA). Generally, DSA was performed at the 6-month and 1-year follow-ups; thereafter, CTA or MRA were performed yearly. Immediate and follow-up angiographic results were classified using the O'Kelly–Marotta (OKM) grading scale (A, total filling; B, subtotal filling; C, entry remnant; D, no filling). Favorable angiographic outcome was defined as OKM grades C and D. Recurrence was defined as any increase in the size of the aneurysmal remnant during follow-up.

### Statistical Analysis

Statistical analyses were performed using SPSS software version 25 (IBM Corp., Armonk, NY, USA). Continuous variables are presented as means with standard deviation. Categorical variables are reported as proportions. The Shapiro–Wilk test was used to assess normality of variables. Patients and aneurysms were grouped according to type of treatment (conventional or FD stent). Group comparisons were performed using the independent samples *t*-test, χ^2^ test, or Fisher exact test as appropriate. *P* < 0.05 was considered significant.

## Results

### Patient and Aneurysm Characteristics

A total of 77 patients with 80 BTVBJ aneurysms underwent EVT: 67 (87%) were treated electively and 10 (13%) in the setting of subarachnoid hemorrhage. Forty-three aneurysms in 40 (57.3%) patients were treated using conventional stents and 34 aneurysms in 34 (42.7%) patients were treated using FDs. Overall, mean patient age was 54.57 ± 13.8 years (range, 10–76). Twenty-five were female (32.5%) and 52 were male (67.5%). Clinical presentation was as follows: incidental finding, 15 (19.5%); acute subarachnoid hemorrhage, 10 (13%); mass effect on the brain stem, 31 (40.3%); ischemic stroke, 7 (9.1%); and headache, 14 (18.2%). Mean aneurysm size was 11.8 ± 8.7 mm (range, 1.2–38.3). Mean clinical follow-up was 27.9 ± 14.9 months. Mean imaging follow-up was 12.3 ± 8.3 months.

Age, sex, smoking, alcohol use, hypertension, diabetes, cardiac disease, and aneurysm location did not significantly differ between the groups. Aneurysm morphology significantly differed between groups (*p* = 0.001): in the FD group, 9 aneurysms (26.5%) were saccular and 25 (73.5%) were fusiform; in the conventional stent group, the corresponding numbers were 30 (65.2%) and 16 (34.8%), respectively. Mean aneurysm diameter (16.4 vs. 8.3 mm; *p* < 0.001) and proportion of aneurysms ≥10 mm in diameter (70.6 vs. 34.8%; *p* = 0.002) were significantly higher in the FD group. A significantly higher proportion of patients in the FD group were symptomatic (91.2 vs. 72.1%; *p* = 0.036). Patient and aneurysm characteristics are shown in Table 1.

**Table 1:** Patient and aneurysm characteristics.

	**FD group**	**Conventional**	**Total**	**Significance**
		**stents group**		**(*P*-Value)**
Patients	34	43	77	
Mean Age (yrs)	50.88 ± 18.6	57.5 ± 7.4	54.57 ± 13.8	0.064
Female, *n* (%)	15 (44.1%)	10 (23.3%)	25 (32.5%)	0.052
Co-morbidities, *n* (%)				
Hypertension	19 (55.9%)	31 (72.1%)	50 (64.9%)	0.139
Diabetes	4 (11.8%)	12 (27.9%)	16 (20.8%)	0.083
Cerebral infarction	7 (20.6%)	16 (37.2%)	23 (29.9%)	0.114
Cardiac disease	3 (8.8%)	7 (16.3%)	10 (13.0%)	0.334
Smoking	11 (32.4%)	22 (51.2%)	33 (42.9%)	0.098
Drinking	8 (23.5%)	19 (44.2%)	27 (35.1%)	0.059
Symptomatic, *n* (%)	31 (91.2%)	31 (72.1%)	62 (80.5%)	**0.036**
Presentation, *n* (%)				
Stroke	1 (2.9%)	6 (14.0%)	7 (9.1%)	
SAH	3 (8.8%)	7 (16.3%)	10 (13.0%)	
Mass effect	18 (52.9%)	13 (30.2%)	31 (40.3%)	
Headache	9 (26.5%)	5 (11.6%)	14 (18.2%)	
Incidental	3 (8.8%)	12 (27.9%)	15 (19.5%)	
Aneurysm	34	46	80	
Mean aneurysm diameter	16.42 ± 10.5	8.34 ± 4.9	11.79 ± 8.7	**0.000**
Aneurysm size				
Small (<10 mm)	10 (29.4%)	30 (65.2%)	40 (50.0%)	**0.002**
Large (10–25 mm)	15 (44.1%)	15 (32.6%)	30 (37.5%)	0.293
Giant (>25 mm)	9 (26.5%)	1 (2.2%)	10 (12.5%)	**0.001**
Location, *n* (%)				0.064
BA trunk	21 (61.8%)	37 (80.4%)	58 (72.5%)	
VB junction	13 (38.2%)	9 (19.6%)	22 (27.5%)	
Morphology, *n* (%)				**0.001**
Saccular	9 (26.5%)	30 (65.2%)	39 (48.8%)	
Fusiform	25 (73.5%)	16 (34.8%)	41 (51.2%)	
Treatment modality				**0.000**
Stents alone	21 (61.8%)	0 (0%)	21 (26.3%)	
Stents with coils	13 (38.2%)	46 (100%)	59 (73.8%)	
Operation time (min)	134.51	133.35	133.86	0.731

*FD, flow diverter; SAH, subarachnoid hemorrhage; BA, basilar artery; VB, vertebrobasilar*.

*Boldface type indicates statistical significance*.

### Postprocedural Angiographic and Clinical Results

EVT was successful in all BTVBJ aneurysms. All parent arteries and relevant branches showed good patency on postoperative angiography. Twenty-one aneurysms (62.5%) in the FD group were treated with stenting alone, compared with no aneurysm in the conventional stent group (*p* < 0.001). The rate of favorable angiographic outcome (OKM grades C and D) immediately after the procedure was significantly lower in the FD group (29.4%vs. 84.8%; *p* < 0.001).

**Figure 2:**
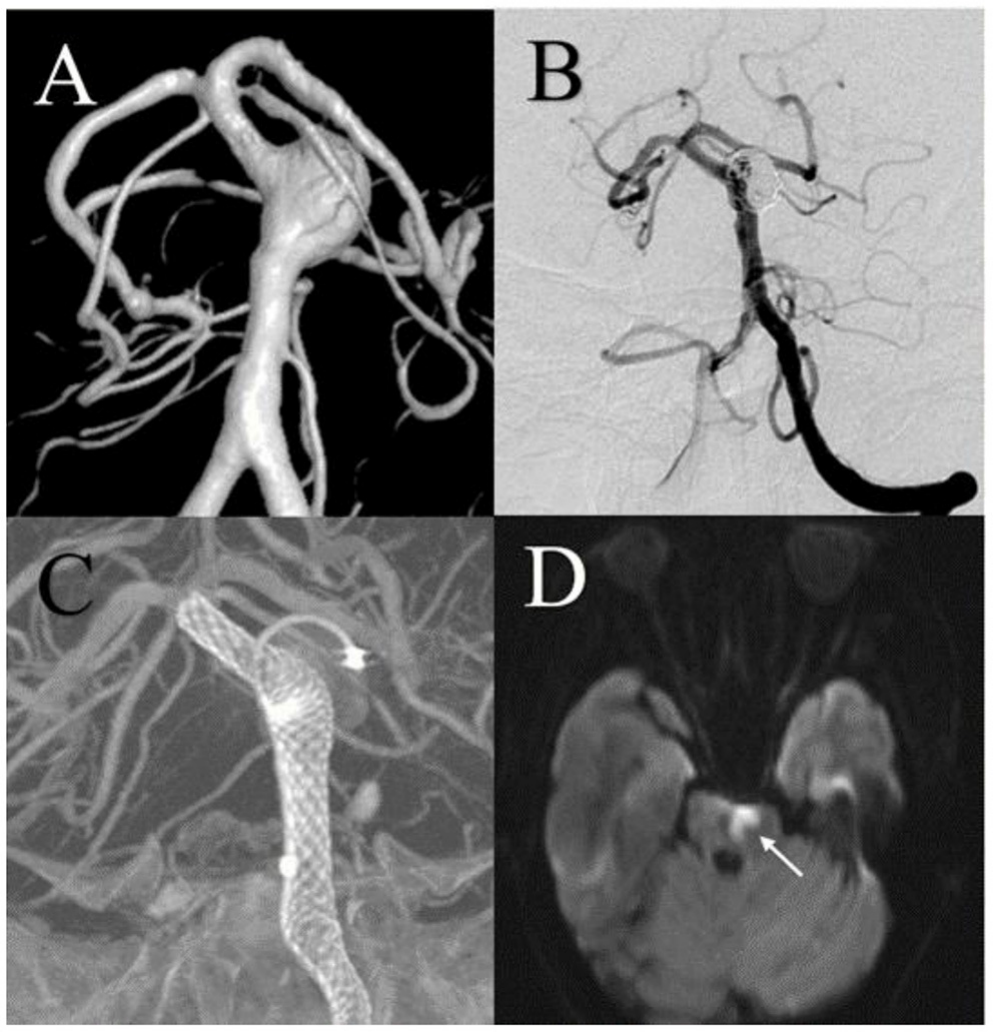
A 68-year-old male with a large basilar trunk aneurysm was treated using a flow diversion stent and experienced an acute ischemic complication. **(A)** Digital subtraction angiography showed the large aneurysm. **(B,C)** Postembolization angiography showed almost complete embolization of the aneurysm. The Pipeline Embolization Device (Medtronic, Minneapolis, MN, USA) exhibited good vessel wall apposition. **(D)** Two days after the procedure, acute ischemic stroke was shown on diffusion-weighted imaging.

**Figure 3:**
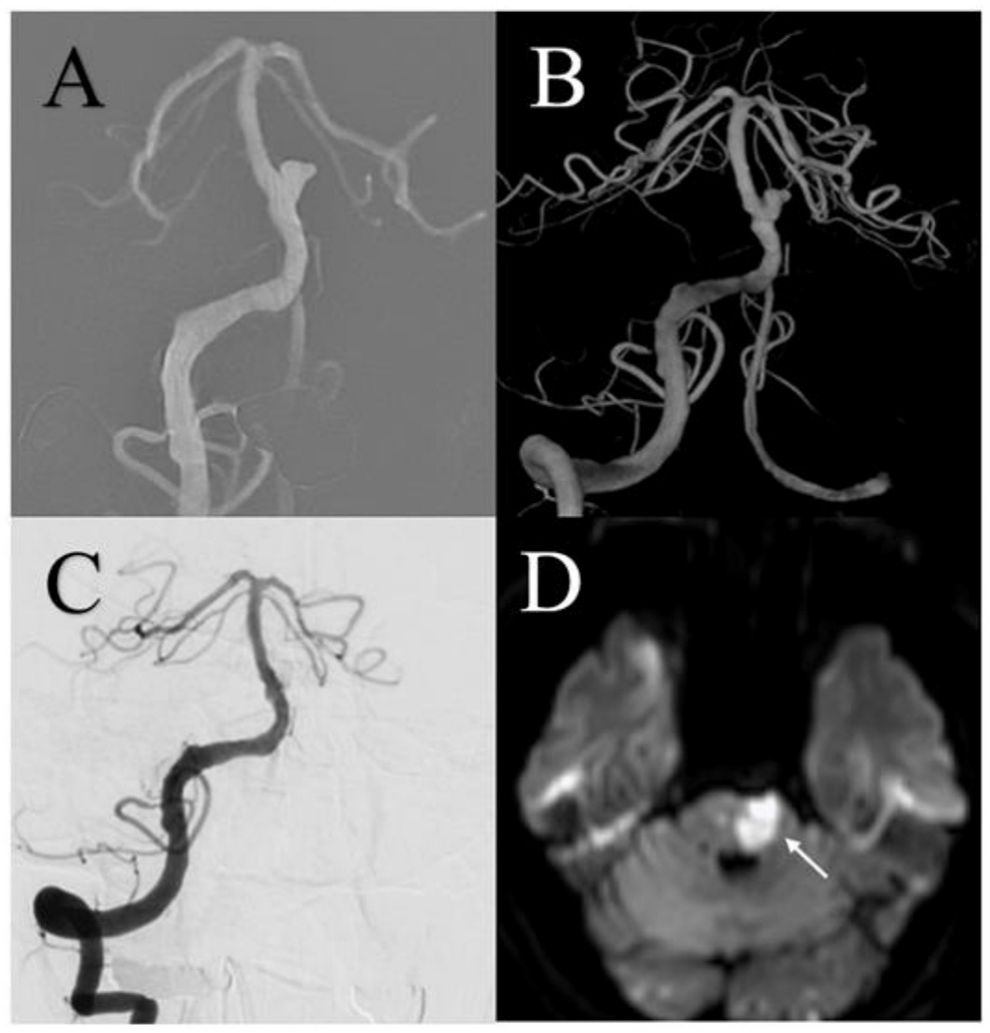
A 61-year-old male with a small basilar trunk aneurysm was treated using an LVIS stent (MicroVention, Tustin, California, USA) and experienced an acute ischemic complication. **(A,B)** Preoperative anteroposterior and three-dimensional reconstruction digital subtraction angiography showed a small aneurysm. **(C)** Angiography immediately after the procedure showed complete aneurysm embolization. **(D)** One week after the procedure, acute ischemic stroke was shown on diffusion-weighted imaging.

Procedure-related complications occurred in 8 patients (23.5%) in the FD group and 10 patients (23.3%) in the conventional stent group (*p* = 0.978). Ischemic events were the most common complication and occurred in 4 patients (11.8%) in the FD group and 9 patients (20.9%) in the conventional stent group (*p* = 0.286; Figures 2, 3). Delayed aneurysmal rupture occurred in 1 patient (2.9%) in the FD group and 2 (4.7%) patients in the conventional stent group (*p* = 0.700). Three patients (8.8%) in the FD group experienced worsening mass effect symptoms after the procedure; all three harbored a giant aneurysm (>25 mm).

Before treatment, mRS score did not significantly differ between groups (*p* = 0.942). The incidence of poor clinical outcome at the time of hospital discharge was 14.7% in the FD group and 16.3% in the conventional stent group. The difference was not significant (*p* = 0.850). Postprocedural angiographic and clinical results are shown in Table 2. Table 3 presents detailed information of the patients who experienced a poor clinical outcome at the time of hospital discharge.

**Table 2:** Immediate and follow-up angiographic and clinical outcomes.

	**FD group**	**Conventional**	**Total**	**Significance**
		**stents group**		**(*P*-Value)**
Immediate angiographic	34	46	80	
Favorable results, *n* (%)	10 (29.4%)	39 (84.8%)	49 (61.3%)	**0.000**
C	4 (11.8%)	16 (34.8%)	20 (25%)	
D	6 (17.6%)	23 (50.0%)	29 (36.3%)	
Unfavorable results, *n* (%)	24 (70.6%)	7 (15.2%)	31 (38.8%)	
A	14 (41.2%)	3 (6.5%)	17 (21.3%)	
B	10 (29.4%)	4 (8.7%)	14 (17.5%)	
Last angiographic	24	35	59	
Favorable results, *n* (%)	19 (79.2%)	27 (77.1%)	46 (78%)	0.854
C	2 (8.3%)	9 (25.7%)	11 (18.6%)	
D	17 (70.8)	18 (51.4%)	35 (59.3%)	
Unfavorable results, *n* (%)	5 (20.8%)	8 (22.9%)	13 (22%)	
A	2 (8.3%)	1 (2.9%)	3 (5%)	
B	3 (12.5%)	7 (20%)	10 (16.9%)	
Change of occlusion, *n* (%)				
Progressive occlusion	15 (62.5%)	2 (5.7%)	17 (28.8%)	**0.000**
Stable occlusion	8 (33.3%)	26 (74.3%)	34 (57.6%)	**0.002**
Recanalization	1 (4.2%)	7 (20%)	8 (13.6%)	0.081
Angiographic follow-up time (Mean, months)	13.1	11.5	12.3	0.58
mRS score at admission, n(%)				0.942
0~2	31 (91.2%)	39 (90.7%)	70 (90.0%)	
3~6	3 (8.8%)	4 (9.3%)	7 (9.1%)	
mRS score at discharge, *n* (%)				0.850
0~2	29 (85.3%)	36 (83.7%)	65 (84.4%)	
3~6	5 (14.7%)	7 (16.3%)	12 (15.6%)	
mRS score at follow-up, *n* (%)	32	40	72	0.888
0~2	25 (73.5%)	31 (72.1%)	56 (72.7%)	
3~6	9 (26.5%)	12 (27.9%)	21 (27.3%)	
Clinical follow-up time (Mean, months)	28.5	27.4	27.9	0.759
Mortality rate, *n* (%)	6 (17.6%)	3 (7.0%)	9 (11.7%)	0.148
Complication, *n* (%)	8 (23.5%)	10 (23.3%)	18 (23.4%)	0.978
BA trunk	5 (23.8%)	8 (21.6%)	13 (22.4%)	0.848
VB junction	3 (23.1%)	2 (22.2%)	5 (22.7%)	1
Second operation, *n* (%)	1 (2.9%)	2 (4.7%)	3 (3.9%)	0.700

*FD, flow diverter; mRS, modified Rankin scale*.

*Boldface type indicates statistical significance*.

**Table 3:** Clinical details in 10 patients who experienced a poor clinical outcome at time of hospital discharge.

**Case No**.	**Age (Y)/ Sex**	**Symptoms**	**Group**	**Location**	**Size (mm)**	**Coils**	**Complications**	**Immediate**	**Last**	**mRS at**	**mRS at**	**mRS at**
								**OKM**	**Last OKM**	**admission**	**discharge**	**last**
1	62/M	Dizziness	2	BT	6.22	Yes	Cerebral infarction/hemiplegia	B	D	1	4	4
2	64/M	TIA	2	BT	10.9	Yes	Cerebral infarction/ coma	B	B	1	4	4
3^#^	58/M	SAH/HH 2	2	BT	10.2 (BT)/8.84 (BT)	Yes	Cerebral infarction/hemiplegia	D/D	D/D	2	5	3
4	65/M	SAH/HH 4	2	BT	12.9	Yes	Hemorrhage/ coma	D	NA	5	4	6
5	58/F	SAH/HH 4	2	VBJ	7.01	Yes	Hemorrhage/ coma	C	B	5	4	2
6	61/M	Dizziness/ diplopia	2	BT	7.52	Yes	Cerebral infarction/hemiplegia	C	NA	2	5	2
7	68/F	Dizziness	1	BT	9.03	Yes	Perforator ischemia/hemiplegia	B	D	1	3	3
8	37/F	Headache	1	BT	31.5	Yes	Stent retraction	A	D	1	3	1
9	12/M	Headache/ diplopia	1	BT	24.7	No	Contrast neurotoxicity/ coma	B	NA	1	6	6
10	49/F	Headache/ dysphagia	1	VBJ	38.3	Yes	Mass effect/ coma	B	NA	2	3	6

# *This patient harbored two aneurysms*.

*Patients 1–6 were treated using conventional stents and patients 7–10 using FD stents*.

*Y, years; F, female; M, male; BT, basilar trunk; VBJ, vertebrobasilar junction; HH, Hunt-Hess grade; SAH, subarachnoid hemorrhage; TIA, transient ischemic attack; mRS, modified Rankin scale; OKM, O'Kelly–Marotta grading scale*.

### Follow-Up Angiographic and Clinical Outcome

Angiographic follow-up was available for 24 patients (70.6%) in the FD group and 35 patients (81.4%) in the conventional stent group. Mean angiographic follow-up was 13.1 ± 10 months in the FD group and 11.5 ± 6 months in the conventional stent group. Favorable angiographic outcome (OKM grades C and D) was achieved at last follow-up in 19 patients (79.2%) in the FD group and 27 patients (77.1%) in the conventional stent group (*p* = 0.854).

Clinical follow-up data were available for all patients. Mean clinical follow-up was 28.5 months (range, 5–67) in the FD group and 27.4 months (range, 5–57) in the conventional stent group. Favorable clinical outcome (mRS score 0–2) was achieved at last follow-up in 25 patients (73.5%) in the FD group and 31 patients (72.1%) in the conventional stent group (*p* = 0.888). Overall mortality was 11.7% (9/77); mortality rates in the FD and conventional stent groups were 17.6% (6/34) and 7.0% (3/43), respectively.

**Figure 4:**
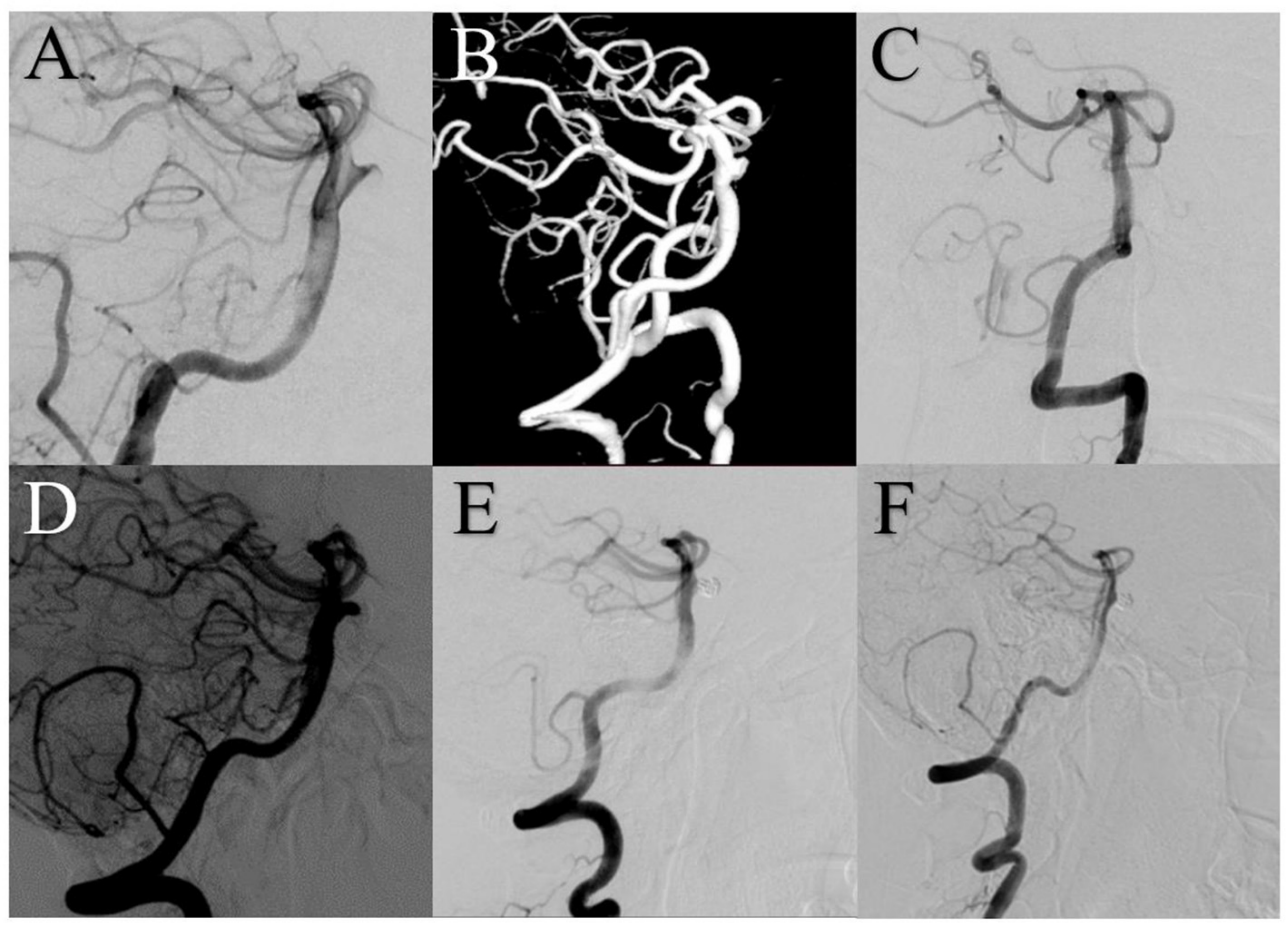
A 44-year-old female with a small basilar trunk aneurysm was treated using an Enterprise stent (Cerenovus, Raynham, Massachusetts, USA). **(A,B)** Digital subtraction angiography (DSA) showed the small aneurysm. **(C)** DSA immediately after treatment showed complete aneurysm embolization. **(D)** Follow-up DSA at 6 months revealed aneurysm recurrence at the level of the neck. **(E)** DSA after coil embolization of the neck remnant showed complete embolization. **(F)** Six months later, DSA showed stable complete occlusion.

In the patients with angiographic follow-up, the recurrence rates were 4.2% (1/24) in the FD group and 20% (7/35) in the conventional stent group (*p* = 0.124). The re-treatment rate was 2.9% (1/34) in the FD group (because of stent migration) and 4.7% (2/43) in the conventional stent group (both because of recanalization) but the difference was not significant (Figure 4). Detailed follow-up outcome data are summarized in Table 2.

### Treatment Results According to Aneurysm Size

BTVBJ aneurysms were divided into two subgroups according to size using a 10 mm cutoff. Incidence of favorable angiographic outcome at last follow-up was significantly higher in patients with aneurysms <10 mm in size (87.9 vs. 65.74%; *p* = 0.038), as was incidence of favorable clinical outcome (83.8 vs. 62.5%; *p* = 0.036). Among patients with aneurysms ≥10 mm, favorable clinical outcome was achieved in 75% (18/24) of patients in the FD group and 43.8% (7/16) of patients in the conventional stent group (*p* = 0.046); the incidence of procedure-related complications was lower in the FD group but the difference was not significant (20.8 vs. 37.5%; *p* = 0.247). Among patients with aneurysms <10 mm, the incidence of favorable clinical outcome was higher (70 vs. 88.9%; *p* = 0.313) and the incidence of procedure-related complications was lower (30 vs. 13.3%; *p* = 0.471) in the conventional stent group; however, the differences were not significant. Treatment results according to aneurysm size are summarized in Table 4.

**Table 4:** Treatment results according to aneurysm size.

	**FD group**	**Conventional**	**Total**	**Significance**
		**stents group**		**(*P*-Value)**
**Aneurysm size≥10 mm**				
Patients, *n* (%)	24 (70.6%)	16 (37.2%)	40 (51.9%)	
Mortality rate, *n* (%)	5 (20.8%)	2 (12.5%)	7 (17.5%)	0.497
Follow-up of angiographic^#^	17	9	26	
Favorable results, *n* (%)	12 (70.6%)	5 (55.6%)	17 (65.4%)	0.667
Unfavorable results, *n* (%)	5 (29.4%)	4 (44.4%)	9 (34.6%)	
Complication, *n* (%)	5 (20.8%)	6 (37.5%)	11 (27.5%)	0.247
Follow-up of clinical outcome	24	16	40	
mRS score, *n* (%)				**0.046**
0~2	18 (75.0%)	7 (43.8%)	25 (62.5%)	
3~6	6 (25.0%)	9 (56.3%)	15 (37.5%)	
**Aneurysm size <10 mm**				
Patients, *n* (%)	10 (29.4%)	27 (62.8%)	37 (48.1%)	
Mortality rate, *n* (%)	1 (10%)	1 (3.7%)	2 (5.4%)	1
Follow-up of angiographic^#^	7	26	33	
Favorable results, *n* (%)	7 (100%)	22 (84.6%)	29 (87.9%)	0.555
Unfavorable results, *n* (%)	0 (0%)	4 (15.4%)	4 (12.1%)	
Complication, *n* (%)	3 (30%)	4 (14.8%)	7 (18.9%)	0.471
Follow-up of clinical outcome	10	27	37	
mRS score, *n* (%)				0.313
0~2	7 (70%)	24 (88.9%)	31 (83.8%)	
3~6	3 (30%)	3 (11.1%)	6 (16.2%)	

# *Angiographic outcome was divided into favorable (O'Kelly–Marotta grading scale C and D) and unfavorable (O'Kelly–Marotta grading scale A and B)*.

*FD, flow diverter; mRS, modified Rankin scale*.

*Boldface type indicates statistical significance*.

## Discussion

The risk of enlargement and rupture is higher in aneurysms located in the posterior circulation, particularly those of the vertebrobasilar junction or basilar trunk (5, 11, 12). Considering the potentially fatal consequences, early intervention is necessary. Surgical treatment' for BTVBJ aneurysms is challenging and associated with high morbidity and mortality because they are deep, difficult to reach, and surrounded by key cerebrovascular structures. Kalani et al. (13) reported only a 27.3% favorable clinical outcome rate in conjunction with 45.4% mortality in patients with large and giant BTVBJ aneurysms who underwent extracranial-intracranial bypass and vessel occlusion. In view of the high rates of disability and mortality associated with surgical treatment of BTVBJ aneurysms, safer and more effective treatments are required, especially for large and giant ones. Traditional EVT modalities have already demonstrated acceptable safety and efficacy profiles; however, outcomes after EVT of large and giant BTVBJ aneurysms remains unsatisfactory (3, 13–16). FDs have become an important tool for treating aneurysms previously considered untreatable (17). However, since perforating arteries are often located near and within BTVBJ aneurysms, many interventionalists still prefer to use traditional techniques rather than FDs. Therefore, evaluating the efficacy of EVT for BTVBJ aneurysms and comparing outcomes between FDs and conventional stenting is warranted.

In a meta-analysis of flow diversion treatment of posterior circulation non-saccular aneurysms, Kiyofuji et al. (18) reported that neurologic outcome differs according to aneurysm location. Vertebral aneurysms were associated with a higher rate of good neurologic outcome than BTVBJ aneurysms. Several studies have reported unsatisfactory outcomes after EVT of BTVBJ aneurysms. Wang et al. (19) reported an 82.1% favorable outcome rate in conjunction with 17.9% mortality. Another study reported a 78.6% satisfactory outcome rate (3). A more recent retrospective study showed a 15% mortality with a favorable clinical outcome rate of only 67.5%; however, the less favorable clinical outcome rate in this study may be due to the fact that 67.5% of patients presented with aneurysmal rupture (16). Two different studies that comprised 10 patients with vertebrobasilar junction aneurysms showed more encouraging results: a favorable clinical outcome was achieved in all patients in both (8, 20). In contrast, Meckel et al. (21) reported a good outcome in only 60% of patients with large or giant vertebrobasilar junction aneurysms. Although the results of EVT for BTVBJ aneurysms have not always been satisfactory, they may be acceptable compared with the results of other treatments. Furthermore, the studies above only described or reported the safety and efficacy of a single method and did not compare different EVTs.

In our study, 56 BTVBJ aneurysm patients overall (72.7%) achieved a favorable clinical outcome after EVT at last follow-up. Forty patients (50%) harbored large or giant aneurysms. Favorable clinical outcome rate did not significantly differ between patients treated using FDs or conventional stents (73.5 vs. 72.1%, *p* = 0.888). This agrees with prior studies that found no difference in clinical or angiographic outcomes among several EVTs (10, 16). However, 20% of aneurysms treated using a conventional stent later recanalized while those treated using a FD stent showed stable progressive occlusion. In the FD group, the initial occlusion rate (OKM grade C and D) was 28.1%, which increased to 79.2% at last follow-up. The initial and follow-up occlusion rates in the conventional stent group were 88.4 and 77.1%, respectively. Angiographic outcome at last follow-up did not significantly differ between the groups; however, incidence of progressive occlusion was significantly higher in the FD group (62.5 vs. 5.7%; *p* < 0.001). Although the procedure-related complication rate in our study was high, it did not significantly differ between the FD (23.5%) and conventional stent groups (23.3%) and is in line with rates reported in other studies (19, 21). The main risk in either group is an ischemic complication. Although FD treatment would appear to be a high-risk approach to BTVBJ aneurysms because of the presence of numerous brainstem perforators, our study found no significant difference in procedure-related complications between the FD and conventional stent groups.

The FD group in our study harbored a higher number of large and giant (70.6%) or fusiform (73.5%) aneurysms, which can cause brain stem mass effect and are associated with a higher risk of brain ischemia and infarction. This explains the significantly higher proportion of symptomatic patients in the FD group (91.2 vs. 72.1%, *p* = 0.036). Although sex did not significantly differ between the groups, the proportion of women was higher in the FD group. This is consistent with previous studies that reported a higher rates of aneurysm growth in women (11).

In our study, the proportions of fusiform and giant aneurysms were higher in the FD group than the conventional stent group. Considering that the natural history of non-saccular giant BTVBJ aneurysms is poor (14, 22, 23), worse results would be expected in the FD group. However, in our subgroup analysis of patients with aneurysms ≥10 mm, the incidence of favorable clinical outcome at last follow-up was significantly higher in the FD group (75 vs. 43.8%; *p* = 0.046) with a lower procedural complication rate (20.8 vs. 37.5%; *p* = 0.247). Most patients in our study had symptoms on admission and 10 (13%) presented with subarachnoid hemorrhage, which may explain the high incidence of complications. Nonetheless, the occlusion rate (OKM grades C and D) did not differ significantly between the groups, despite a higher occlusion rate in the FD group at last follow-up (70.6 vs. 55.6%; *p* = 0.667). For patients with aneurysms <10 mm, the favorable clinical outcome rate did not significantly differ between the conventional stent group (88.9%) and FD group (70%), nor did the procedure-related complication rate (14.8 and 30%, respectively) or occlusion rate (84.6 and 100%, respectively). The same analyses were performed for fusiform aneurysms but no significant differences were observed, possibly because of small sample size. Our results lead us to conclude that FD stents are superior to conventional stents for the treatment of large and giant BTVBJ aneurysms. However, for smaller aneurysms, both FD stents and conventional stents are feasible and effective.

## Study Limitations

This study has several limitations. First, it was retrospective in nature and performed in a single center; therefore, both selection and treatment bias may have been introduced. Second, long-term angiographic follow-up results were not available in all patients and we did not have detailed data for patients who died. Third, mean imaging follow-up was 12.3 months, which is too short to determine the rate of final complete embolization; the difference in occlusion rate between the FD and conventional stent groups may have been significant if patient follow-up was longer. Finally, our cohort was small, as BTVBJ aneurysms are rare. Future large-scale studies are warranted to confirm our findings.

## Conclusion

EVT of small BTVBJ aneurysms (<10 mm) using either FDs or conventional stents was feasible and effective. FDs achieved a higher occlusion rate and more favorable clinical outcome at last follow-up in patients with large or giant aneurysms (≥10 mm). Future large-scale studies with long-term follow-up are warranted to determine the best EVT for BTVBJ aneurysms.

## Data Availability Statement

The original contributions presented in the study are included in the article/supplementary material, further inquiries can be directed to the corresponding authors.

## Ethics Statement

The studies involving human participants were reviewed and approved by Institutional Review Board of Beijing Tiantan Hospital. The patients/participants provided their written informed consent to participate in this study.

## Author Contributions

QP collected the clinical data, performed the statistical analysis, and wrote the manuscript. YZho, WL, CW, and LD helped collect the clinical data. SM and YZha helped revise the manuscript, designed the research, and handled funding and supervision. All authors read and approved the final manuscript.

## Funding

This work was supported by the National Natural Science Foundation of China (Grant Numbers: 81801158 and 81571128).

## Conflict of Interest

The authors declare that the research was conducted in the absence of any commercial or financial relationships that could be construed as a potential conflict of interest.

## Publisher's Note

All claims expressed in this article are solely those of the authors and do not necessarily represent those of their affiliated organizations, or those of the publisher, the editors and the reviewers. Any product that may be evaluated in this article, or claim that may be made by its manufacturer, is not guaranteed or endorsed by the publisher.
